# Association between frequency of spicy food consumption and hypertension: a cross-sectional study in Zhejiang Province, China

**DOI:** 10.1186/s12986-021-00588-7

**Published:** 2021-07-06

**Authors:** Hao Wang, Lingli Chen, Dun Shen, Yuan Cao, Xiaoyi Zhang, Kaixu Xie, Chunmei Wang, Shuiqing Zhu, Pei Pei, Yu Guo, Fiona Bragg, Min Yu, Zhengming Chen, Liming Li

**Affiliations:** 1 Department of NCDs Control and Prevention, Zhejiang Provincial Center for Diseases Control and Prevention, #3399 Binsheng Road, Binjiang District, Hangzhou, Zhejiang Province China; 2 Department of NCDs Control and Prevention, Tongxiang City Center for Disease Control and Prevention, Tongxiang, China; 3 Chinese Academy of Medical Sciences, Beijing, China; 4 Medical Research Council Population Health Research Unit, Nuffield Department of Population Health, University of Oxford, Oxford, UK; 5 Department of Epidemiology and Biostatistics, School of Public Health, Peking University Health Science Center, Beijing, China; 6 Peking University Center for Public Health and Epidemic Preparedness and Response, Beijing, China

**Keywords:** Hypertension, Spicy food, Cross-sectional study

## Abstract

**Background:**

Hypertension is a known risk factor for multiple chronic diseases. Existing literature on the association between frequency of spicy food consumption and hypertension shows mixed findings.

**Methods:**

The analyses are based on the Tongxiang baseline dataset of the China Kadoorie Biobank prospective study, including data from electronic questionnaires, physical measurements and blood sample collection. A total of 53,916 participants aged 30–79 years were included in the final analysis. Multivariable logistic regression was used to estimate the association of spicy food consumption with hypertension, and multiple linear regression was performed to explore the association of spicy food consumption with systolic and diastolic blood pressure.

**Results:**

Of the 53,916 participants, 23,921 had prevalent hypertension. 12.3% of participants reported consuming spicy food weekly. Among female participants, after adjusting for socio-demographic status, lifestyle factors, BMI, waist circumference, sleep duration and snoring, when compared with females who never consumed spicy food, the odds ratios (95% CI) for hypertension were 1.02 (0.96–1.08), 0.90 (0.79–1.01), and 0.88 (0.78–0.99), respectively, for females who consumed spicy food less than once weekly, 1–2 times weekly, and ≥ 3 times weekly (*P*_*trend*_ = 0.04). The corresponding odds ratios for males were 1.02 (0.95–1.09), 1.07 (0.95–1.20), and 0.91 (0.81–1.01), respectively (*P*_*trend*_ = 0.39). Among current alcohol drinkers, compared to participants who never consumed spicy food, the odds ratio (95% CI) for hypertension among participants consuming spicy food daily was 0.98 (0.80–1.20). The corresponding figure for non-current drinkers was 0.72 (0.62–0.84). The association was stronger among non-current alcohol drinkers than among current drinkers (*P*_*heterogeneity*_ = 0.02).

**Conclusions:**

Frequency of spicy food consumption is inversely associated with hypertension in females, but not in males.

**Supplementary Information:**

The online version contains supplementary material available at 10.1186/s12986-021-00588-7.

## Background

Hypertension is one of the main causes of cardiovascular diseases [1, 2]. High systolic blood pressure is the leading risk factor for death in China, and was estimated to account for 2.54 million deaths in 2017, of which 95.7% were due to cardiovascular diseases [3]. Recently, a nationally representative survey, including 451,755 adults over 18 years of age from 31 provinces in mainland China, indicated that 23.2% (≈ 244.5 million) of Chinese adults had hypertension, and 41.3% (≈ 435.3 million) had prehypertension [4]. Hence, hypertension remains a serious public health problem in China.

Worldwide, spices are an essential part of culinary cultures, with a long history of use for flavoring, coloring and preserving food, as well as for medicinal purposes [5, 6]. Spices are recognized as one of the primary tastes in ancient Asia, especially in India and China [7, 8].

In past decades, the effects of spicy food consumption on chronic diseases and other conditions, including obesity, cancer, ischemic heart disease, cerebrovascular disease, fracture, dyslipidemia and impaired cognitive function, have been studied [9–15]. Some studies suggest lower disease risks associated with consumption of spicy food [11, 12, 14], while others suggest disease risks are higher [9, 10, 15]. The association between spicy food and hypertension has received considerable attention in recent years. However, the results from existing studies are similarly controversial [12, 16–18]. For example, while Ahuja and colleagues found that four weeks of regular chilli consumption had no obvious effects on blood pressure among healthy free-living individuals [16], Tingchao et al. reported that frequency of spicy food consumption was inversely associated with risk of hypertension in women [12]. Hence, the aim of this study was to examine the association of frequency of spicy food consumption with hypertension using data from the CKB study in Tongxiang, Zhejiang.

## Methods

### Study population and design

Detailed information about the CKB study design, survey methods and population has been described elsewhere [19–21]. The data utilized in the current study were obtained from Tongxiang, one of the 10 regions included in the CKB study. In brief, 57,704 participants aged 30–79 years were recruited and participated in the baseline survey between August 2004 and January 2008. The baseline survey consisted of a questionnaire, physical measurements and blood sample collection. All survey operations were conducted by trained, qualified staff using standardized procedures. For the current study, participants who had a history of cancer (n = 163), stroke (n = 349), heart disease (n = 464), physician-diagnosed diabetes (n = 1380), or baseline screen-detected diabetes (n = 1432) were excluded. After these exclusions, a total of 53,916 (22,573 men, 31,343 women) participants remained for inclusion in the final analyses.

### Outcome variable

Blood pressure was measured in a seated position at least twice using a digital sphygmomanometer (Omron UA-779) in a quiet room with constant indoor temperature around 20 °C. Two measurements were undertaken with a 5-min interval between measurements. If the difference between the first systolic blood pressure (SBP) and second SBP measurement was greater than 10 mm Hg, a third measurement was conducted and average of the last two measurements was recorded and used for analyses [1]. Participants were considered to be hypertensive if they had a measured SBP ≥ 140 mm Hg, a measured diastolic blood pressure (DBP) ≥ 90 mm Hg, or reported a prior history of doctor-diagnosed hypertension or use of antihypertensive medication.

### Assessment of exposure variable

Frequency of spicy food consumption was assessed through the question “how often did you eat hot spicy food during the past month?”. Answer options included: “Never or almost never” (i.e., non-consumers), “only occasionally” (i.e., less than once weekly), “1–2 days/week”, “3–5 days/week”, and “daily or almost every day”. In analyses, those who chose “3–5 days/week” or “daily or almost every day”” were combined into one group (i.e., ≥ 3 days/week). The reproducibility of frequency of spicy food consumption was tested twice with a median interval of 1.4 years. Spearman’s coefficient for the correlation was 0.71, indicating that spicy food consumption was reported consistently [11].

### Assessment of covariates

An interviewer-administered electronic questionnaire included socio-demographic characteristics (age, sex, education level, marital status, and household income), lifestyle factors (cigarette smoking, alcohol drinking, physical activity, fresh fruit intake, meat intake, sleep duration and snoring), personal medical history (cancer, stroke, diabetes, and heart attack), family history of hypertension, and, among women, menopause status.

Cigarette smoking and alcohol drinking were categorized into four groups according to participants’ responses: (1) non-smokers (or non-drinkers); (2) former smokers (or former drinkers); (3) occasional smokers (or occasional drinkers); and (4) current smokers (or current drinkers) [22, 23]. Total physical activity was converted into metabolic equivalent of task hours per day (MET-hours/day) based on transportation, occupation, housework, and non-sedentary recreation as described in previous studies [24, 25].

Physical measurements included height, weight, and waist circumference (WC), measured using calibrated instruments by trained health workers. Standing height was measured to the nearest 0.1 cm with a stadiometer. Weight was measured to the nearest 0.1 kg with a body composition analyzer. Body mass index (BMI) was calculated as weight in kilograms divided by the square of standing height in meters, and obesity was defined as BMI ≥ 25.0 kg/m^2^ [26]. WC was measured to the nearest 0.1 cm with a non-stretchable tape measure at the midpoint between the lowest rib and the iliac crest. Excessive WC was defined as ≥ 85 cm for males, and ≥ 80 cm for females [27]. A non-fasting venous blood sample was collected. Immediate on-site testing of plasma glucose level was undertaken.

### Statistical analysis

Statistical analyses were performed using SAS 9.4 (SAS Institute Inc., Cary, NC, USA). Descriptive statistics were presented as mean ± SD or percentages for continuous or categorical variables, respectively. To examine the association between frequency of spicy food consumption and risk of hypertension, univariate and multivariable logistic regression analyses were applied. Participants who never consumed spicy food were considered as the reference group. Potential confounding factors, including socio-demographic status and lifestyle factors were adjusted for in different models. In model 1, odds ratios were adjusted for age (continuous) and sex. Model 2 included additional adjustment for education level (no formal school, primary school, middle school, and high school or above) and household income (≤ 19,999 yuan, 20,000–34,999 yuan, ≥ 35,000 yuan). Model 3 included additional adjustment for cigarette smoking (never, occasional, former, and current), alcohol drinking (never, occasional, former, and current), physical activity (continuous), meat consumption (daily and non-daily), fruit consumption (daily and non-daily), BMI (continuous), WC (continuous), snoring (never, occasional, and habitual) and sleep duration (continuous). Multiple linear regression analyses were further performed to explore the associations of frequency of spicy food consumption with systolic blood pressure and diastolic blood pressure. Stratified analyses were conducted to detect whether the associations of daily spicy food consumption with prevalent hypertension differed according to age (30–49 y or 50–79 y), education level (illiterate or primary, and above), household income (< 35,000 yuan or ≥ 35,000 yuan), physical activity (< 30 MET-h/d or ≥ 30 MET-h/d), smoking status (current smokers or non-current smokers), alcohol status (current drinkers or non-current drinkers), meat consumption (daily or non-daily), fruit consumption (daily or non-daily), BMI (< 25 kg/m^2^ or ≥ 25 kg/m^2^), WC (normal or excessive), sleep duration (< 7.6 h/d or ≥ 7.6 h/d), or menopause status (menopausal or non-menopausal). In sensitivity analyses, 1128 participants with self-reported physician-diagnosed peptic ulcer disease were excluded from the analyses. All statistical significance was set at *P* = 0.05.

## Results

### Characteristics of participants

Of the 53,916 participants, 23,921 had prevalent hypertension. The prevalence of hypertension among participants who consumed spicy food never, less than once per week, 1–2 times weekly, and ≥ 3 times weekly were 64.0%, 23.7%, 5.8% and 6.5%, respectively.

Compared with non-consumers, participants who consumed spicy food frequently were more likely to be younger, male, well-educated, current smokers, current drinkers, habitual snorers, physically inactive, to consume meat and fruit frequently, to have higher BMI and WC, and to report longer sleep duration. No significant difference was found in household income (*P* = 0.51) or in menopause status (*P* = 0.65) according to frequency of spicy food consumption (Table 1).

**Table 1:** Baseline characteristics of participants according to frequency of spicy food consumption

Characteristics	Overall (N = 53,916)	Frequency of spicy food consumption				*P*_*trend*_
		Never (N = 34,513)	< 1 times/week (N = 12,781)	1–2 times/week (N = 3109)	≥ 3 times/week (N = 3513)	
Mean age (years)^a^	52.5 ± 9.9	53.8 ± 9.9	50.8 ± 9.4	48.6 ± 8.8	48.5 ± 9.0	< 0.0001
Females (%)^b^	58.1	62.2	53.2	47.3	43.4	< 0.0001
No formal education (%)	43.5	44.5	41.1	39.5	41.9	< 0.0001
Income ≥ 35,000 (yuan) (%)	37.8	36.2	43.8	33.1	34.6	0.51
Current smokers (%)	28.0	26.9	29.1	29.7	31.3	< 0.0001
Current drinkers (%)	17.1	14.1	20.0	22.8	26.9	< 0.0001
Physical activities (MET-h/d)	30.6 ± 15.3	30.6 ± 15.2	31.0 ± 15.2	30.7 ± 15.0	30.2 ± 15.9	0.0024
Consuming meat daily (%)	15.2	13.2	16.5	20.4	24.5	< 0.0001
Consuming fruit daily (%)	6.7	5.6	6.7	11.6	14.6	< 0.0001
BMI (kg/m^2^)	22.9 ± 3.1	22.8 ± 3.1	23.0 ± 3.1	23.0 ± 3.0	23.3 ± 3.1	< 0.0001
WC (cm)	76.5 ± 9.1	76.3 ± 9.0	76.9 ± 9.0	76.6 ± 9.1	77.1 ± 9.3	< 0.0001
Habitual snoring (%)	24.3	23.4	26.0	25.3	26.7	< 0.0001
Sleep duration (hours)	7.6 ± 1.2	7.6 ± 1.2	7.6 ± 1.1	7.8 ± 1.1	7.7 ± 1.2	< 0.0001
Women with menopause (%)^b,c^	53.9	54.0	53.0	52.5	52.6	0.65

Data were adjusted for age and sex unless specified

*MET* metabolic equivalent of task, *BMI* body mass index, *WC* waist circumference

^a^Adjusted for sex

^b^Adjusted for age

^c^Only in women

### Association of frequency of spicy food consumption with hypertension

In univariate analysis, compared to non-consumers, odds ratios (95% CI) for hypertension among participants who consumed spicy food less than once per week, 1–2 times per week, and ≥ 3 times per week were 0.89 (0.86–0.93), 0.77 (0.71–0.83), and 0.74 (0.69–0.79), respectively.

After adjustment for age, sex, education level, and household income, participants who consumed spicy food less than once per week, 1–2 times per week, and ≥ 3 times per week had odds ratios (95% CI) for hypertension of 1.05 (1.01–1.10), 1.03 (0.95–1.11), and 0.97 (0.90–1.04), respectively, when compared with non-consumers. After further adjustment for cigarette smoking, alcohol drinking, physical activity, meat intake, fruit intake, BMI, WC, self-reported snoring, and sleep duration, the associations were attenuated, with corresponding odds ratios (95% CI) of 1.01 (0.97–1.06), 0.98 (0.91–1.07), and 0.88 (0.82–0.96), respectively. When the association was explored separately among women, the corresponding odds ratios (95% CI) were 1.02 (0.96–1.08), 0.90 (0.79–1.01), and 0.88 (0.78–0.99), respectively (*P*_*trend*_ = 0.04). However, such an inverse association was not found among males (*P*_*trend*_ = 0.39) (Table 2).

**Table 2:** Unadjusted and adjusted odds ratios for hypertension associated with frequency of spicy food consumption among adults in Zhejiang

Frequency of spicy food intake	N. participants	Univariate	Multivariable		
			Model 1	Model 2	Model 3
Total					
Never	34,513	1.00	1.00	1.00	1.00
< 1 times/week	12,781	0.89 (0.86–0.93)	1.06 (1.01–1.10)	1.05 (1.01–1.10)	1.01 (0.97–1.06)
1–2 times/week	3109	0.77 (0.71–0.83)	1.02 (0.94–1.10)	1.03 (0.95–1.11)	0.98 (0.91–1.07)
≥ 3 times/week	3513	0.74 (0.69–0.79)	0.96 (0.89–1.04)	0.97 (0.90–1.04)	0.88 (0.82–0.96)
*P*_*trend*_		< 0.0001	0.86	0.74	0.02
Males^a^					
Never	13,211	1.00	1.00	1.00	1.00
< 1 times/week	5900	0.92 (0.87–0.98)	1.10 (1.03–1.17)	1.08 (1.01–1.15)	1.02 (0.95–1.09)
1–2 times/week	1581	0.89 (0.80–0.99)	1.17 (1.05–1.30)	1.17 (1.05–1.31)	1.07 (0.95–1.20)
≥ 3 times/week	1881	0.83 (0.75–0.92)	1.05 (0.95–1.16)	1.05 (0.95–1.16)	0.91(0.81–1.01)
*P*_*trend*_		< 0.0001	0.02	0.02	0.39
Females^a^					
Never	21,302	1.00	1.00	1.00	1.00
< 1 times/week	6881	0.85 (0.80–0.90)	1.03 (0.97–1.09)	1.03 (0.97–1.09)	1.02 (0.96–1.08)
1–2 times/week	1528	0.62 (0.56–0.69)	0.87 (0.78–0.98)	0.89 (0.79–0.99)	0.90 (0.79–1.01)
≥ 3 times/week	1632	0.60 (0.54–0.67)	0.88 (0.79–0.98)	0.90 (0.80–1.00)	0.88 (0.78–0.99)
*P*_*trend*_		< 0.0001	0.02	0.06	0.04

In model 1, odds ratios were adjusted for age (continuous) and sex. Model 2 included additional adjustment for education level (no formal school, primary school, middle school, and high school or above), household income (≤ 19,999 yuan, 20,000–34,999 yuan, ≥ 35,000 yuan), Model 3 included additional adjustment for cigarettes consumption (never, occasional, former, and current), alcohol consumption (never, occasional, former, and current), physical activity (continuous), meat consumption (daily and non-daily), fruit consumption (daily and non-daily), BMI (continuous), WC (continuous), snoring (never, occasional, and habitual), sleep duration (continuous)

^a^Without adjustment for sex

### Association of frequency of spicy food consumption and blood pressure

After adjusting for potential confounders in model 3 (age, sex, socio-demographic and lifestyle factors, adiposity, snoring and sleep duration), compared with non-consumers, the adjusted *β* coefficients (95% CI) for systolic blood pressure associated with consumption of spicy food < 1 times/week, 1–2 times/week, and ≥ 3 times/week were 0.31 (− 0.08, 0.71), − 0.41 (− 1.13, 0.31), and − 0.96 (− 1.64, − 0.27), respectively (*P*_*trend*_ < 0.05). When the association was examined separately among women, the corresponding *β* coefficients (95% CI) were 0.46 (− 0.08, 0.99), − 0.37 (− 1.39, 0.64), and − 1.06 (− 2.06, − 0.07), respectively (*P*_*trend*_ < 0.05) i.e., there was an inverse association between frequency of spicy food consumption and systolic blood pressure among females. However, there was no statistically significant trend observed among males (*P*_*trend*_ = 0.24) (Table 3).

**Table 3:** Unadjusted and adjusted *β* coefficients for systolic blood pressure associated with frequency of spicy food consumption among adults in Zhejiang

	Frequency of spicy food consumption				*P*_*trend*_
	Never	< 1 times/week	1–2 times/week	≥ 3 times/week	
N. participants (total)	34,513	12,781	3109	3513	
SBP, mmHg	135.9 ± 21.4	134.8 ± 20.8	132.6 ± 20.2	132.6 ± 20.7	
Unadjusted β (95% CI)	Ref	− 1.14 (− 1.57, − 0.71)	− 3.39 (− 4.16, − 2.61)	− 3.36 (− 4.09, − 2.62)	< 0.0001
Model 1 adjusted β (95% CI)	Ref	0.73 (0.32, 1.14)	− 0.20 (− 0.94, 0.55)	− 0.30 (− 1.00, 0.41)	0.88
Model 2 adjusted β (95% CI)	Ref	0.74 (0.32, 1.15)	− 0.06 (− 0.81, 0.69)	− 0.21 (− 0.92, 0.49)	0.63
Model 3 adjusted β (95% CI)	Ref	0.31 (− 0.08, 0.71)	− 0.41 (− 1.13, 0.31)	− 0.96 (− 1.64, − 0.27)	0.03
N. participants (males)^a^	13,211	5900	1581	1881	
SBP, mmHg	137.0 ± 20.8	136.3 ± 19.9	134.8 ± 19.7	135.5 ± 20.4	
Unadjusted β (95% CI)	Ref	− 0.74 (− 1.37, − 0.11)	− 2.24 (− 3.31, − 1.17)	− 1.55 (− 2.54, − 0.56)	< 0.0001
Model 1 adjusted β (95% CI)	Ref	1.02 (0.40, 1.63)	0.52 (− 0.52, 1.56)	0.84 (− 0.12, 1.80)	0.01
Model 2 adjusted β (95% CI)	Ref	0.94 (0.32, 1.55)	0.61 (− 0.43, 1.65)	0.87 (− 0.09, 1.84)	0.01
Model 3 adjusted β (95% CI)	Ref	0.19 (− 0.40, 0.79)	− 0.43 (− 1.44, 0.57)	− 0.60 (− 1.54, 0.34)	0.24
N. participants (females)^a^	21,302	6881	1528	1632	
SBP, mmHg	135.3 ± 21.8	133.5 ± 21.5	130.3 ± 20.4	129.3 ± 20.6	
Unadjusted β (95% CI)	Ref	− 1.74 (− 2.33, − 1.15)	− 5.01 (− 6.13, − 3.89)	− 6.01 (− 7.09, − 4.92)	< 0.0001
Model 1 adjusted β (95% CI)	Ref	0.52 (− 0.04, 1.08)	− 0.92 (− 1.98, 0.14)	− 1.44 (− 2.47, − 0.41)	0.03
Model 2 adjusted β (95% CI)	Ref	0.59 (0.04, 1.15)	− 0.69 (− 1.75, 0.37)	− 1.23 (− 2.27, − 0.20)	0.12
Model 3 adjusted β (95% CI)	Ref	0.46 (− 0.08, 0.99)	− 0.37 (− 1.39, 0.64)	− 1.06 (− 2.06, − 0.07)	0.03

In model 1, odds ratios were adjusted for age (continuous) and sex. Model 2 included additional adjustment for education level (no formal school, primary school, middle school and high school or above), household income (≤ 19,999 yuan, 20,000–34,999 yuan, ≥ 35,000 yuan), Model 3 included additional adjustment for cigarettes consumption (never, occasional, former, and current), alcohol consumption (never, occasional, former, and current), physical activity (continuous), meat consumption (daily and non-daily), fruit consumption (daily and non-daily), BMI (continuous), WC (continuous), snoring (never, occasional, and habitual snoring), sleep duration (continuous)

^a^Without adjustment for sex

Similar to the association with systolic blood pressure, frequency of spicy food consumption was negatively related to diastolic blood pressure among females (*P*_*trend*_ < 0.05), but no significant association was observed among males (*P*_*trend*_ = 0.38) (Table 4).

**Table 4:** Unadjusted and adjusted *β* coefficients for diastolic blood pressure associated with frequency of spicy food consumption among adults in Zhejiang

	Frequency of spicy food consumption				*P*_*trend*_
	Never	< 1 times/week	1–2 times/week	≥ 3 times/week	
N. participants (total)	34,513	12,781	3109	3513	
DBP, mmHg	80.3 ± 10.7	80.6 ± 10.8	80.3 ± 10.7	80.5 ± 11.1	
Unadjusted β (95% CI)	Ref	0.30 (0.09, 0.52)	0.06 (− 0.34, 0.45)	0.20 (− 0.17, 0.57)	0.08
Model 1 adjusted β (95% CI)	Ref	0.21 (− 0.01, 0.43)	− 0.09 (− 0.48, 0.31)	− 0.01 (− 0.38, 0.36)	0.75
Model 2 adjusted β (95% CI)	Ref	0.18 (− 0.03, 0.40)	− 0.02 (− 0.41, 0.38)	0.04 (− 0.33, 0.41)	0.55
Model 3 adjusted β (95% CI)	Ref	− 0.04 (− 0.26, 0.17)	− 0.23 (− 0.61, 0.15)	− 0.39 (− 0.75, − 0.02)	0.03
N. participants (males)^a^	13,211	5900	1581	1881	
DBP, mmHg	81.5 ± 11.0	82.0 ± 11.0	81.8 ± 11.0	82.1 ± 11.5	
Unadjusted β (95% CI)	Ref	0.45 (0.11, 0.79)	0.30 (− 0.27, 0.87)	0.65 (0.11, 1.18)	0.004
Model 1 adjusted β (95% CI)	Ref	0.46 (0.11, 0.80)	0.31 (− 0.27, 0.89)	0.66 (0.12, 1.19)	0.004
Model 2 adjusted β (95% CI)	Ref	0.36 (0.02, 0.71)	0.36 (− 0.22, 0.94)	0.68 (0.14, 1.22)	0.004
Model 3 adjusted β (95% CI)	Ref	− 0.07 (− 0.40, 0.27)	− 0.24 (− 0.80, 0.32)	− 0.16 (− 0.68, 0.36)	0.38
N. participants (females)^a^	21,302	6881	1528	1632	
DBP, mmHg	79.5 ± 10.4	79.4 ± 10.4	78.8 ± 10.3	78.6 ± 10.3	
Unadjusted β (95% CI)	Ref	− 0.11 (− 0.40, 0.17)	− 0.70 (− 1.24, − 0.16)	− 0.97 (− 1.49, − 0.44)	< 0.0001
Model 1 adjusted β (95% CI)	Ref	0.02 (− 0.26, 0.31)	− 0.45 (− 0.99, 0.09)	− 0.69 (− 1.22, − 0.16)	0.01
Model 2 adjusted β (95% CI)	Ref	0.05 (− 0.23, 0.34)	− 0.35 (− 0.89, 0.19)	− 0.59 (− 1.12, − 0.06)	0.04
Model 3 adjusted β (95% CI)	Ref	− 0.03 (− 0.30, 0.25)	− 0.25 (− 0.78, 0.28)	− 0.57 (− 1.08, − 0.05)	0.04

In model 1, *β* coefficients were adjusted for age (continuous) and sex. Model 2 included additional adjustment for education level (no formal school, primary school, middle school and high school or above), household income (≤ 19,999 yuan, 20,000–34,999 yuan, ≥ 35,000 yuan), Model 3 included additional adjustment for cigarettes consumption (never, occasional, former, and current), alcohol consumption (never, occasional, former, and current), physical activity (continuous), meat consumption (daily and non-daily), fruit consumption (daily and non-daily), BMI (continuous), WC (continuous), snoring (never, occasional, and habitual snoring), sleep duration (continuous)

^a^Without adjustment for sex

### Subgroup analyses

In subgroup analyses, the strength of the association between frequency of spicy food consumption and hypertension was largely consistent across subgroups defined by age, education level, household income, physical activity, cigarette smoking, meat consumption, fruit consumption, BMI, WC, sleep duration, and menopause status (*P*_*heterogeneity*_ > 0.05). However, there was a significantly stronger association among non-current alcohol drinkers (OR = 0.72, 95% CI, 0.62–0.84) than among current alcohol drinkers (OR = 0.98, 95% CI, 0.80–1.20) (*P*_*heterogeneity*_ = 0.02) (Table 5).

**Table 5:** Adjusted odds ratios for hypertension associated with consuming spicy food daily versus never according to participant characteristics

	N. hypertension	ORs (95% CI)	*P*_*heterogeneity*_
Age group (years)			0.67
30–49	4139	0.76 (0.64–0.90)	
50–79	12,222	0.72 (0.61–0.86)	
Education level			0.51
No formal education	8739	0.86 (0.70–1.04)	
Primary or above	7622	0.79 (0.67–0.92)	
Household income			0.69
< 35,000 (yuan)	10,602	0.80 (0.69–0.93)	
≥ 35,000 (yuan)	5759	0.84 (0.68–1.04)	
Physical activity (MET-h/d)			0.06
< 30	9344	0.72 (0.61–0.86)	
≥ 30	7017	0.91 (0.77–1.08)	
Smoking status			0.14
Current smokers	4059	0.90 (0.75–1.08)	
Non-current smokers	12,302	0.75 (0.63–0.88)	
Alcohol status			0.02
Current drinkers	2654	0.98 (0.80–1.20)	
Non-current drinkers	13,707	0.72 (0.62–0.84)	
Meat consumption			0.41
Daily	2006	0.75 (0.60–0.94)	
Non-daily	14,355	0.84 (0.73–0.97)	
Fruit consumption			0.59
Daily	795	0.88 (0.63–1.22)	
Non-daily	15,566	0.80 (0.70–0.91)	
BMI (kg/m^2^)			0.34
< 25	11,260	0.86 (0.74–0.99)	
≥ 25	5101	0.76 (0.61–0.94)	
WC			0.97
Normal	7103	0.81 (0.67–0.97)	
Excessive	9258	0.81 (0.69–0.95)	
Sleep duration (hours/day)			0.29
< 7.6	7377	0.75 (0.61–0.91)	
≥ 7.6	8984	0.85 (0.73–0.99)	
Menopause status			0.94
Menopausal	6841	0.80 (0.59–1.08)	
Non-menopausal	2777	0.79 (0.61–1.01)	

Odds ratios were adjusted for age (continuous) and sex, education level (no formal school, primary school, middle school and high school or above), household income (≤ 19,999 yuan, 20,000–34 999 yuan, ≥ 35,000 yuan), cigarettes consumption (never, occasional, former, and current), alcohol consumption (never, occasional, former, and current), physical activity (continuous), meat consumption (daily and non-daily), fruit consumption (daily and non-daily), BMI (continuous), WC (continuous), snoring (never, occasional, and habitual), sleep duration (continuous)

*ORs* odds ratios, *CI* confidence intervals, *BMI* body mass index, *WC* waist circumference

### Sensitivity analyses

In sensitivity analyses, after exclusion of 1128 participants with self-reported physician-diagnosed peptic ulcer disease, there was no marked change in the association of frequency of spicy food consumption with hypertension. Among female participants, compared to non-consumers, odds ratios (95% CI) for hypertension among those who consumed spicy food less than once per week, 1–2 times per week, and ≥ 3 times per week were 1.02 (0.96–1.09), 0.89 (0.79–1.01), and 0.88 (0.78–0.99), respectively (*P*_*trend*_ = 0.04). The corresponding figures for males were 1.01 (0.95–1.09), 1.08 (0.96–1.21), and 0.92 (0.83–1.03), respectively (*P*_*trend*_ = 0.39) (Additional file 1: Table S1).

## Discussion

This large cross-sectional study explored the association of spicy food consumption with prevalent hypertension. Frequent spicy food consumption was inversely associated with hypertension in females. However, such an inverse association was not found in males.

### Frequency of spicy food consumption

A previously published paper from the CKB study indicated that 99.7% of participants in Hunan consumed spicy food weekly, while only 8.8% of participants in Haikou consumed spicy food weekly [9]. In the present study, the prevalence of weekly spicy food consumption was 12.3%, much lower than in the CKB population as a whole, in which 42.5% of participants consumed spicy food weekly [9]. This discrepancy reflects geographic distribution of Chinese residents' preferences for spicy food. Zhejiang is located in the east of China, and compared with residents living in most other provinces, residents in Zhejiang have a preference for a more bland diet rather than heavy tastes.

Consistent with previous studies [12, 28], participants who more frequently consumed spicy food were more likely to be young, male, current smokers, alcohol drinkers, and to more frequently eat meat and fruit. In contrast with an earlier study, indicating that individuals with high levels of spicy food consumption had lower BMI levels when compared with non-consumers [17], the present study documented that individuals who consumed spicy food more frequently seemed to have higher BMI and WC than non-consumers. This may reflect investigation of crude BMI values, without adjustment for age. Sun et al. found that spicy food consumption was positively associated with adiposity, including both general and abdominal obesity [9], similar with the current study. This positive association may reflect increased palatability of meals including spicy food [7].

### Relationship of frequency of spicy food consumption with hypertension

Harada et al. found that SBP and DBP were significantly lower among hypertensive volunteers after administration of a mixture of capsaicin and isoflavone for 5 months, but not among normotensive volunteers [18]. In a randomized cross-over dietary intervention study from Australia, 36 individuals (22 women and 14 men) consumed a chilli diet (30 g chilli per day) and a bland diet (chilli-free) for 4 weeks each [16]. This study concluded that four weeks of regular chilli consumption had no obvious beneficial or harmful effects on SBP or DBP, which was inconsistent with the present study. However, a prospective cohort involving 13,670 Chinese adults [17], with a median follow-up of 9.0-years, demonstrated that, after adjustment for age, gender, energy, sodium and fat intake, smoking, alcohol, and physical activity, when compared with non-consumers, the HRs (95% CI) for incident hypertension among participants who consumed chilli in quantities of 1–20 g/day, 20.1–50 g/day and ≥ 50.1 g/day were 0.80 (0.73–0.88), 0.81 (0.73–0.89) and 0.65 (0.57–0.75), respectively, consistent with the current study.

The 2009 China Health and Nutrition Survey (CHNS), including 9273 adults aged ≥ 18 years old, was conducted in nine geographically diverse provinces. Findings from the CHNS indicated that, compared with females who did not eat spicy food, the adjusted odds ratios (95% CI) of hypertension for women who consumed spicy food 1–2 times/week, 3–4 times/week, and ≥ 5 times/week were 0.92 (0.78–1.09), 0.91 (0.73–1.13), and 0.74 (0.57–0.96), respectively, but this inverse association was not found in men [12]. This sex disparity in the association between frequency of spicy food consumption and hypertension was compatible with the current study.

Two large prospective population studies conducted in China and Italy illustrated that spicy food intake was associated with a lower risk of death due to cardiovascular diseases [11, 14]. High blood pressure is a well-known risk factor for cardiovascular diseases [1, 2], and the inverse association between spicy food consumption and hypertension found in the current study suggests the apparent protective effect of spicy food on cardiovascular disease mortality may be mediated via lowering of blood pressure.

In subgroup analyses, significant differences in the association of spicy food consumption with hypertension were observed across strata of alcohol consumption, with a stronger inverse association among non-current drinkers than current drinkers. Intriguingly, prospective analyses based on 0.48 million CKB participants showed that the inverse association of spicy food consumption with all-cause mortality was stronger in participants who did not drink alcohol than among those who did drink alcohol [11], which is in line with the current study.

Although mechanisms underlying the potential beneficial effect of spicy food consumption on hypertension have not yet been fully elucidated, several hypotheses have been proposed. First, capsaicin, as the major pungent element in red pepper, is a neurotoxic agent, and could activate transient receptor potential vanilloid type-1 (TRPV1), in turn improving endothelium-dependent vasorelaxation and lowering blood pressure [29]. Second, activation of TRPV1 could reduce vascular lipid accumulation and attenuate atherosclerosis [30]. Third, activation of TRPV1 may prevent adipogenesis and obesity [31]. Lastly, enjoyment of spicy food may significantly reduce individual salt preference, daily salt intake, and blood pressure by modifying the neural processing of salty taste in the brain [32].

The findings of the current study are of potential clinical and public health importance. Firstly, spicy food might be a valuable dietary intervention for prevention of hypertension in both healthy populations and high-risk groups, especially in regions with typically low consumption of spicy food, such as Zhejiang. Secondly, spicy food should not be considered a dietary taboo for patients with hypertension. However, further evidence from prospective and randomized studies will be important in establishing this potential clinical relevance.

## Strengths and limitations

The strengths of this study include a large sample size, use of standardized data collection procedures, and strict control for established and potential risk factors for hypertension. Several limitations merit mention, however. First, the cross-sectional design restricts establishment of the temporal relationship of spicy food consumption with hypertension. Second, assessment of spicy food consumption in the current study was self-reported and subject to measurement error. Third, although multiple established and potential risk factors for hypertension were adjusted for in different models, residual confounding by other unmeasured or unknown biological and social factors is still possible.

## Conclusions

In conclusion, the current study shows that frequency of spicy food consumption is inversely associated with hypertension in females, but not in males.

## Supplementary Information

**Additional file 1: Table S1.** Unadjusted and adjusted odds ratios for hypertension associated with frequency of spicy food consumption among adults without peptic ulcer disease in Zhejiang.

## Data Availability

Details of how to access China Kadoorie Biobank data and details of the data release schedule are available from www.ckbiobank.org/site/Data+Access.

## Abbreviations

CKB: China Kadoorie Biobank
SBP: Systolic blood pressure
DBP: Diastolic blood pressure
MET: Metabolic equivalent tasks
BMI: Body mass index
WC: Waist circumference
ORs: Odds ratios
CI: Confidence interval
HRs: Hazard ratios
CHNS: China health and nutrition survey
TRPV1: Transient receptor potential vanilloid type-1
