# Supplementary material for: Association between frequency of spicy food consumption and hypertension: a cross-sectional study in Zhejiang Province, China
Source: Nutr Metab (Lond). 2021 Jul 6;18:70. doi: 10.1186/s12986-021-00588-7 (PMC8259443; doi:10.1186/s12986-021-00588-7)
Supplement: Supplementary file 1 — Additional file 1: Table S1. Unadjusted and adjusted odds ratios for hypertension associated with frequency of spicy food consumption among adults without peptic ulcer disease in Zhejiang. [file 12986_2021_588_MOESM1_ESM.docx]

**Table S1 Unadjusted and adjusted odds ratios for hypertension associated with frequency of spicy food consumption among adults without** [**peptic ulcer**](https://www.hujiang.com/ciku/peptic_ulcer/) **disease in Zhejiang**

| Frequency of spicy food consumption | N．participants | Univariate | Multivariable | | |
| --- | --- | --- | --- | --- | --- |
|  |  |  | Model 1 | Model 2 | Model 3 |
| Total |  |  |  |  |  |
| Never | 33 864 | 1.00 | 1.00 | 1.00 | 1.00 |
| < 1 times/week | 12 440 | 0.89 (0.86-0.93) | 1.07 (1.02-1.12) | 1.07 (1.02-1.11) | 1.01 (0.97-1.06) |
| 1-2 times/week | 3 046 | 0.77 (0.71-0.83) | 1.04 (0.96-1.12) | 1.04 (0.96-1.13) | 0.98 (0.90-1.07) |
| ≥ 3 times/week | 3 438 | 0.74 (0.69-0.80) | 0.99 (0.92-1.07) | 0.99 (0.93-1.08) | 0.89 (0.82-0.96) |
| *P _trend_* |  | <0.0001 | 0.24 | 0.21 | 0.03 |
| Males ^a^ |  |  |  |  |  |
| Never | 12 856 | 1.00 | 1.00 | 1.00 | 1.00 |
| < 1 times/week | 5 689 | 0.92 (0.87-0.98) | 1.09 (1.02-1.17) | 1.08 (1.01-1.15) | 1.01 (0.95-1.09) |
| 1-2 times/week | 1 545 | 0.89 (0.80-0.99) | 1.17 (1.05-1.31) | 1.18 (1.05-1.31) | 1.08 (0.96-1.21) |
| ≥3 times/week | 1 832 | 0.84 (0.76-0.93) | 1.06 (0.96-1.18) | 1.07 (0.96-1.18) | 0.92 (0.83-1.03) |
| *P _trend_* |  | <0.0001 | 0.02 | 0.02 | 0.39 |
| Females ^a^ |  |  |  |  |  |
| Never | 21 008 | 1.00 | 1.00 | 1.00 | 1.00 |
| < 1 times/week | 6 751 | 0.85 (0.80-0.90) | 1.03 (0.97-1.09) | 1.04 (0.98-1.10) | 1.02 (0.96-1.09) |
| 1-2 times/week | 1 501 | 0.62 (0.55-0.69) | 0.87 (0.78-0.98) | 0.88 (0.79-0.99) | 0.89 (0.79-1.01) |
| ≥ 3 times/week | 1 606 | 0.60 (0.54-0.67) | 0.88 (0.79-0.98) | 0.89 (0.80-0.99) | 0.88 (0.78-0.99) |
| *P _trend_* |  | <0.0001 | 0.02 | 0.05 | 0.04 |

In model 1, odds ratios were adjusted for age (continuous) and sex. Model 2 included additional adjustment for education level (no formal school, primary school, middle school and high school or above), household income (≤19,999 yuan, 20 000-34 999 yuan, ≥35 000 yuan), Model 3 included additional adjustment for cigarettes consumption (never, occasional, former, and current), alcohol consumption (never, occasional, former, and current), physical activity (continuous), meat consumption (daily and non-daily), fruit consumption (daily and non-daily), BMI (continuous), WC (continuous), snoring (never, occasional, and habitual snoring), sleep duration (continuous).

a: without adjustment for sex.
